# The Predictive Value of Serum ACE2 and TMPRSS2 Concentrations in Patients with COVID-19—A Prospective Pilot Study

**DOI:** 10.3390/jpm12040622

**Published:** 2022-04-12

**Authors:** Reut Kassif Lerner, Michal Stein Yeshurun, Rina Hemi, Nahid Zada, Keren Asraf, Ram Doolman, Stefanie W. Benoit, Maria Helena Santos de Oliveira, Giuseppe Lippi, Brandon Michael Henry, Itai M. Pessach, Naomi Pode Shakked

**Affiliations:** 1 Department of Pediatric Intensive Care, The Edmond and Lily Safra Children’s Hospital, Sheba Medical Center, Tel Hashomer 52621, Israel; reutkl@gmail.com (R.K.L.); itai.pessach@sheba.health.gov.il (I.M.P.); 2 Sackler Faculty of Medicine, Tel-Aviv University, Tel-Aviv 69978, Israel; michal.stein4@gmail.com (M.S.Y.); rina.hemi@sheba.health.gov.il (R.H.); ram.doolman@sheba.health.gov.il (R.D.); 3 Department of Pediatrics, The Edmond and Lily Safra Children’s Hospital, Sheba Medical Center, Tel Hashomer 52621, Israel; 4 Division of Endocrinology and Metabolism, Sheba Medical Center, Tel Hashomer 52621, Israel; nahid.zada@sheba.health.gov.il; 5 The Dworman Automated-Mega Laboratory, Sheba Medical Center, Tel Hashomer 52621, Israel; keren.asraf@sheba.health.gov.il; 6 Division of Pediatric Nephrology and Hypertension, Cincinnati Children’s Hospital Medical Center, Cincinnati, OH 45229, USA; stefanie.benoit@cchmc.org (S.W.B.); brandon.henry@cchmc.org (B.M.H.); 7 Department of Pediatrics, University of Cincinnati College of Medicine, Cincinnati, OH 45267, USA; 8 Biostatistics Master’s Program, State University of Maringa, Maringa 87020-900, Brazil; mariaholiveira34@gmail.com; 9 Section of Clinical Biochemistry, University of Verona, 37134 Verona, Italy; giuseppe.lippi@univr.it

**Keywords:** COVID-19, ACE2, TMPRSS2, respiratory failure, renal failure

## Abstract

One of the major challenges for healthcare systems during the Coronavirus-2019 (COVID-19) pandemic was the inability to successfully predict which patients would require mechanical ventilation (MV). Angiotensin-Converting Enzyme 2 (ACE2) and TransMembrane Protease Serine S1 member 2 (TMPRSS2) are enzymes that play crucial roles in SARS-CoV-2 entry into human host cells. However, their predictive value as biomarkers for risk stratification for respiratory deterioration requiring MV has not yet been evaluated. We aimed to evaluate whether serum ACE2 and TMPRSS2 levels are associated with adverse outcomes in COVID-19, and specifically the need for MV. COVID-19 patients admitted to an Israeli tertiary medical center between March--November 2020, were included. Serum samples were obtained shortly after admission (day 0) and again following one week of admission (day 7). ACE2 and TMPRSS2 concentrations were measured with ELISA. Of 72 patients included, 30 (41.6%) ultimately required MV. Serum ACE2 concentrations >7.8 ng/mL at admission were significantly associated with the need for MV (*p* = 0.036), inotropic support, and renal replacement therapy. In multivariate logistic regression analysis, elevated ACE2 at admission was associated with the need for MV (OR = 7.49; *p* = 0.014). To conclude, elevated serum ACE2 concentration early in COVID-19 illness correlates with respiratory failure necessitating mechanical ventilation. We suggest that measuring serum ACE2 at admission may be useful for predicting the risk of severe disease.

## 1. Introduction

Coronavirus Disease 2019 (COVID-19), caused by severe acute respiratory syndrome coronavirus 2 (SARS-CoV-2), is responsible for the most severe public health crisis of the last century. As of October 2021, the World Health Organization (WHO) has documented over 250 million confirmed COVID-19 cases and 5 million deaths worldwide.

One of the major effectors challenging healthcare systems and clinicians worldwide is the inability to effectively predict who will require intensive care support or mechanical ventilation to enable early intervention, proper risk stratification, and allocation of limited hospital resources. Thus, finding a way to predict the disease course of patients with COVID-19 has been the focus of many studies [1,2,3,4,5,6].

Similar to its phylogenetic counterpart, SARS-CoV, two enzymes have been shown to regulate the process of SARS-CoV-2 entry into host cells. The viral spike protein binds to Angiotensin Converting Enzyme 2 (ACE2), a component of the Renin-Angiotensin-Aldosterone System (RAAS) and undergoes a first cleavage. The second is TransMembrane Protease Serine S1 member 2 (TMPRSS2), a serine protease expressed by human epithelial cells in the airway, gastrointestinal tract, prostate and additional organs, which cleaves the SARS-CoV-2 spike protein subunits 1 and 2, thus enabling the direct fusion of S2 subunit with the targeted cell membrane. It is comprised of a transmembrane domain, a receptor domain, a scavenger receptor cysteine-rich domain and a protease domain. The protease domain was shown to be cleaved and secreted; however, the normal range for serum levels has yet to be established. TMPRSS2 has been put forward as an essential component for H1N1 influenza virus (as well as other influenza strains) entry into the cell, via cleavage of the viral HemAgglutinins (HAs) [7,8,9]. Additionally, it was demonstrated to facilitate the entry of several viruses into host cells via cleaving of the viral envelope glycoproteins. These include the Influenza virus, the human coronaviruses (i.e., HCoV-229E, MERS-CoV, SARS-CoV and most recently, SARS-CoV-2) and several strains of parainfluenza [10]. Inhibition of TMPRSS2 activity has been suggested as a promising approach against viral infection [11]. For example, Camostat mesylate, a protease inhibitor that inhibits TMPRSS2 and is approved for use in other clinical indications, was shown to block SARS-CoV-2 entry into primary human lung cells [12,13]. In addition, cell lines expressing TMPRSS2 were shown to be highly susceptible to SARS-CoV-2 infection [14]. Thus, both ACE2 and TMPRSS2 are required for SARS-CoV-2 infection of human cells.

Both the high rate of SARS-CoV-2 infectivity and respiratory manifestations of COVID-19 in humans can be attributed to the fact that ACE2 is expressed in the apical membrane of the airways and alveoli, as well as endothelial cells of blood vessels and other tissues and organs [15]. Apart from its membrane-bound form, ACE2 also undergoes shedding and thus can be measured in serum in soluble form. The expression of ACE2 is particularly high in endothelial and type 2 alveolar epithelial cells, therefore, mediating entry of SARS-CoV-2 to the respiratory tract system, causing respiratory manifestations following inhalation of airborne droplets and aerosol.

Circulatory ACE2 levels have previously been shown to be higher in males, older individuals, and those with cardiovascular morbidities or inflammatory conditions [16,17,18]. In fact, ACE2 levels have been suggested as a biomarker for adverse cardiac events in a variety of cardiovascular conditions, including heart failure, aortic stenosis, and coronary artery disease [19,20,21]. Additionally, overexpression of human ACE2 in a mouse model of SARS-CoV brought enhanced disease severity [22]. However, while ACE2 and TMPRSS2 have both been suggested as putative therapeutic targets for COVID-19 [13,23,24], their circulatory levels have only recently and scarcely been evaluated as potential biomarkers for disease severity and progression.

Herein we investigated the prognostic value of serum ACE2 and TMPRSS2 levels in COVID-19 patients, to assess whether their measurement at an early stage of disease may predict associated the need for mechanical ventilation and additional unfavorable outcomes.

## 2. Methods

### 2.1. Patient Enrollment

Seventy-two adult patients aged ≥18 years with COVID-19 diagnosed with nasopharyngeal swabs admitted to the largest tertiary medical center in Israel between March 2020 and November 2020 were included in this study. As the Israeli vaccination campaign against SARS-CoV-2 began on 19 December 2020, these patients were not vaccinated at the time of enrollment and sample collection. SARS-CoV-2 RNA was confirmed in all patients by reverse-transcriptase polymerase chain reaction (RT-PCR) assay. Inclusion criteria were admission to our medical center within the study period and RT-PCR confirmation of SARS-CoV-2 infection; patients under 18 years of age were excluded. The study was conducted in accordance with the tenets of the Declaration of Helsinki, and with the approval of the Institutional Review Board (IRB) at the Sheba Medical Center (IRB approval No. SMC-20-7120). The study was deemed no greater than minimal risk and thus performed under a waiver of informed consent.

### 2.2. Sample Collection and Handling

Residual serum samples of COVID-19 patients were collected and centrifuged at 4000× *g* for 5 min at room temperature and maintained at −80 °C until measurement. Human serum Angiotensin Converting Enzyme 2 (ACE2) and Human Transmembrane Protease, Serine 2 (TMPRSS2) levels were measured using enzyme-linked immune sorbent assays (ELISA) (Elabscience, Houston, TX, USA). The sensitivity of the ACE2 assay was 0.23 ng/mL and the inter-assay coefficients of variation (CV) ranged from 4.26 to 5.42%. The sensitivity of the TMPRSS2 assay was 0.10 ng/mL and the inter-assay CV ranged from 4.75 to 5.45%.

Serum concentrations of both ACE2 and TMPRSS2 were analyzed on two occasions: within 24 h of admission to the ward (either Coronavirus Critical Care Unit (CCCU) or general COVID-19 ward) (designated as day 0, early sample), and after 7 days from admission (late sample). Samples were excluded from the study if there was missing data, insufficient serum, or poor serum quality.

### 2.3. Data Collection

Patient clinical data were extracted from the electronic medical records from hospital admission until death or discharge. Patients’ vital signs, including temperature, heart rate, respiratory rate, invasive blood pressure, oxygen (O_2_) saturation, and end tidal carbon monoxide (CO_2_), were continuously monitored. In order to define acute lung injury, a widely accepted tool is the P/F ratio = Pao_2_/fraction of inspired oxygen (FiO_2_), however, this requires arterial blood gas sampling. SpO_2_/FiO_2_ (S/F) has been previously validated as noninvasive criteria for diagnosing lung injury as the change in Pao_2_ correlates well with changes in pulse oximetric saturation (SpO_2_) [25,26]. Therefore, in this study, S/F (saturation/FiO_2_ support) ratio upon presentation to the Emergency Department (ED) was documented (as a surrogate for P/F ratio while avoiding arterial blood sampling) as a value representing the respiratory status and reflecting disease severity upon presentation, and prior to MV and/or admission to the in-patient setting. For calculation of S/F, all patients underwent measurement of SpO_2_ with documentation of inhaled concentrations of oxygen.

Demographic characteristics included age, gender, weight, and ethnic background. Clinical data included co-morbidities (Hypertension, dyslipidemia, diabetes, chronic obstructive pulmonary disease (COPD), malignancy, ischemic heart disease (IHD), chronic renal failure, cerebrovascular accident (CVA)), medications, laboratory results, hospitalization course, and complications.

In addition, laboratory markers were obtained from the patient medical records and were performed as part of their routine testing. These included markers of inflammation (CRP, PCT, WBC, ANC, TNF, IL8, IL6, IL-1B), coagulopathy (D-dimer, fibrinogen), acute kidney injury (creatinine), acute cardiac injury (troponin), liver failure (AST, ALT, ALP, GGT, bilirubin), triglycerides, and routine CBC. Not all lab tests were performed on all patients.

Further data related to the course of hospitalization included admission to a general COVID-19 internal medicine ward versus CCCU, length of stay, respiratory support needed, laboratory results, and complications including disseminated intravascular coagulopathy (DIC), pulmonary embolism (PE)/deep vein thrombosis (DVT), Heparin-induced thrombocytopenia (HIT), bleeding, pneumothorax or sepsis.

### 2.4. Outcomes

The primary aim of this study was to evaluate the predictive value of serum ACE2 and TMPRSS2 concentrations for requiring mechanical ventilation (MV) in patients with COVID-19. The primary outcome was the need for MV. The decision to intubate was based upon clinical judgment and signs of respiratory failure as accepted in the literature for COVID-19 patients [27]. Secondary outcomes included: in-hospital mortality; CCCU admission, prolonged hospitalization, defined as the length of stay of over 7 days; the need for inotropic support; acute kidney injury (AKI), defined as ≥50% increase in creatinine from baseline (according to the KDIGO guidelines [28]); need for renal replacement therapy (RRT); need for extracorporeal membrane oxygenation (ECMO) support, and blood products transfusion.

### 2.5. Statistical Analysis

Categorical variables were reported in frequencies and percentages, and their significance was assessed using the chi-square test or Fischer’s exact test. Shapiro–Wilk test was used to determine the normal distribution of continuous variables. Normally distributed variables were reported as mean and standard deviation values, and their significance was assessed using the student’s *t*-test, while not normally distributed variables were reported as the median and interquartile range (IQR, 25th–75th percentiles), and their significance was assessed using the Mann–Whitney U test. All statistical tests were 2-sided, and a *p*-value of less than 0.05 was considered significant. 

Receiver operating characteristic (ROC) curves were used to determine the best possible cutoff value of serum ACE2 and TMPRSS2 levels for the prediction of the need for mechanical ventilation, based on the sensitivity and specificity calculated for each possible cutoff value. The area under the curve (AUC) was calculated to evaluate the ability of the two biomarkers to discriminate between patients who required MV and those who did not.

Logistic regression analysis was carried out separately for ACE2 and TMPRSS2, to assess an independent association between biomarkers and respiratory failure, adjusting for possible confounders.

Additionally, the ACE2/TMPRSS2 ratio was calculated as the value of ACE2/value of TMPRSS2 in each patient, and also correlated with mentioned outcomes, in order to evaluate the interaction between the two enzymes and to assess if the balance between these enzymes has any clinical significance.

The statistical analysis was carried out using SPSS (version 27, IBM Corp., Armonk, NY, USA), and R software Version 1.4.1103 (The R Foundation, Vienna, Austria) and PyCharm community edition, V2020.1.1 (JetBrains).

## 3. Results

### 3.1. Patient Characteristics

**Figure 1:**
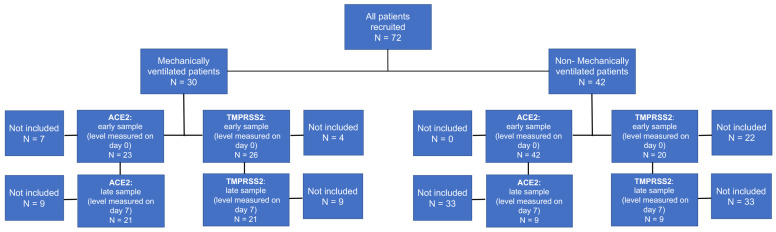
Flow chart of COVID-19 patients recruited for the study. ACE2, Angiotensin converting enzyme 2; TMPRSS2, transmembrane serine protease 2.

A final number of 72 patients with COVID-19 participated in the study. Overall, we analyzed 65 samples for ACE2 early samples and 30 for ACE2 late samples (six with no matching early sample). Forty-six samples were instead analyzed for TMPRSS2 early samples and 30 for late samples (three with no matching early sample) (Figure 1).

The mean age of the cohort was 64.4 (±14.5) years with a predilection for male over female gender (73.7% vs. 26.3%, respectively). Regarding comorbidities, 54.1% of the patients had hypertension, 38.8% diabetes, and 37.5% dyslipidemia. Median serum ACE2 and TMPRSS2 levels at presentation did not significantly differ between patients with or without hypertension (6.24 vs. 5.59 ng/mL and 6.92 vs. 5.52, respectively; *p* = 0.602 and *p* = 0.698) or between those with or without diabetes (6.60 vs. 5.27 ng/mL and 6.07 vs. 5.81, respectively; *p* = 0.446 and *p* = 0.572). Significantly higher median ACE2 levels at presentation were found in patients with dyslipidemia compared to those without (9.15 vs. 4.13 ng/mL; *p* = 0.002), whereas median TMPRSS2 levels did not show significant differences (8.34 vs. 4.75; *p* = 0.099) (Supplementary Table S1).

Patients were stratified according to those who ultimately required intubation and MV due to respiratory failure (MV group; n = 30, 41.6%), and those who did not (non-MV group; n = 42, 58.3%). Both groups had similar demographics and shared the most clinical background characteristics. Interestingly, older age and chronic renal failure were more predominant within the non-MV group (Table 1). SpO_2_/FiO_2_ (S/F) ratio was evaluated for each patient upon presentation to the ED and compared between the two groups. Mean values were 447.6 and 428.6 for non-MV and MV groups, respectively, and multivariate analysis revealed no statistically significant independent association between the measure and intubation (*p* = 0.090 when adjusted for TMPRSS2 and *p* = 0.063 when adjusted for ACE2).

The need for MV was associated with a more complicated disease course demonstrated by increased mortality, prolonged hospitalization, higher complication rates, such as sepsis and renal failure, as well as the need for advanced interventions including ECMO support (Supplementary Table S2). Furthermore, MV was associated with derangements in laboratory values, mainly white blood cell (WBC) count and platelets, as well as increased inflammatory/coagulopathy markers (procalcitonin, IL-6, D-dimer and fibrinogen). Moreover, significantly higher levels of triglycerides upon admission, as well as higher cardiac troponin levels, were seen in patients who ultimately required MV. Further analysis of inflammatory markers, including CPK, TNF, IL-8 and IL-1B did not find significant differences between the groups (Supplementary Table S3).

### 3.2. Increased Circulatory ACE2 Levels in COVID-19 Patients at Early Disease Stages Correlate with an Increased Risk for Intubation and Mechanical Ventilation

Both ACE2 and TMPRSS2 serum levels at presentation were higher in the MV compared to the non-MV patient group (Supplementary Figure S1).

Patients admitted to the CCCU had higher serum ACE2 concentrations at presentation than those who did not: 9.84 (1.13–13.88) vs. 6.7 (0.13–8.15) ng/mL (*p* = 0.038). Furthermore, ACE2 levels at presentation were higher in patients who required RRT for AKI: 13.72 (10.48–16) vs. 7.5 (−0.13–9.99) ng/mL (*p* = 0.018). No association with sepsis or in-hospital mortality was found.

Receiver operating characteristic (ROC) analysis for ACE2 levels at presentation resulted in an AUC of 0.612 (95% CI: 0.465–0.759, *p* = 0.138) and determined a cutoff level of 7.8 ng/mL for MV (Supplementary Figure S2). Considering the sample prevalence of 41.7% intubation, the cutoff also provided a positive predictive value (PPV) of 58.6% and a negative predictive value (NPV) of 68.3%, with 47.8% sensitivity and 73.8% specificity. When assessing the association of serum ACE2 levels at presentation with patients’ outcomes using this cutoff, ACE2 levels above the cutoff were significantly associated with a need for MV (52.2% vs. 26.2%, *p* = 0.036), inotropic support (52.2% vs. 23.8%, *p* = 0.021), RRT (26.1% vs. 4.8%, *p* = 0.012) and with bleeding (39.1% vs. 14.3%, *p* = 0.023) (Table 2).

In multivariate logistic regression analysis, sex, age, dyslipidemia, and acute kidney injury were considered as possible confounders as they were significantly associated with MV in univariate analysis. Serum ACE2 levels above the 7.8 ng/mL cutoff were associated with MV, with an OR of 7.49 (95%CI, 1.51–37.11, *p* = 0.014). Additional parameters independently associated with MV were age, with an OR of 0.89 (95%CI, 0.83–0.95, *p* = 0.001) and AKI with an OR of 17.68 (95%CI, 3.13–99.86, *p* = 0.001) (Table 3). All other parameters were not significantly associated with MV in multivariate logistic regression analysis.

ACE2 levels at the second time point (day 7–10) did not significantly differ between the two groups (*p* = 0.298).

### 3.3. Elevated Early Sample ACE2 Expression Is Significantly Associated with Biomarkers for Severe Disease Course in COVID-19 Patients

Spearman’s correlation coefficient was used to quantify the association between various laboratory values and early sample serum ACE2 expression. We found a significant positive correlation with WBC at admission, as well as with its minimum and maximum values, low platelet count, higher CRP at admission and peak levels, PCT at admission and peak levels, ALT and AST, LDH, along with admission and peak D-dimer levels. There was also a significant and negative correlation between ACE2 and lymphocyte percentage (Supplementary Table S4).

### 3.4. Increased Circulatory TMPRSS2 Levels in COVID-19 Patients at Admission Do Not Significantly Correlate the Risk for Intubation and Mechanical Ventilation

ROC analysis of TMPRSS2 resulted in an AUC of 0.608 (95% CI: 0.433–0.782, *p* = 0.215) determined a cutoff of 2 ng/mL. Considering the sample prevalence of 41.7% MV, the cutoff provided a PPV of 54.5% and NPV of 88.8%, respectively, with 45% specificity and 92.3% sensitivity. Early sample TMPRSS2 value higher than the cutoff was associated with intubation and MV (*p*-value = 0.005). However, in multivariate logistic regression analysis adjusted for sex, age and AKI, this association was lost (Supplementary Table S5).

Higher serum TMPRSS2 levels later in the disease course (days 7–10) were found to be associated with intubation: median level of 6.53 (2.66–20.11) vs. 1.97 (0.54–4.03) ng/mL in patients who required MV and those who did not, respectively; (*p* = 0.045) as well as with CCCU admission: 13.61 (4.26–13.88) ng/mL for patients requiring CCCU admission vs. 3.66 (2.30–7.85) ng/mL in those who did not (*p* = 0.045) (Supplementary Table S6). However, as the sample size of patients for whom these later TMPRSS2 samples were available was small, we were unable to further characterize this association using multivariate analysis.

### 3.5. ACE2/TMPRSS2 Ratio Is Not Significantly Associated with the Risk for Intubation and Mechanical Ventilation

Median ACE2/TMPRSS2 ratio values did not significantly differ between patients who required MV and those who did not, neither was the ratio a significant predictor in multivariate analysis (Supplementary Table S7).

There was also no significant association between the ratio and other outcomes, including AKI, Dialysis, or CCCU admission.

## 4. Discussion

In this study, we sought to ascertain whether the serum concentrations of ACE2 and TMPRSS2 soon after hospital admission may be associated with the need for MV in adult COVID-19 patients. The rationale behind this hypothesis was the crucial role that each of the two proteins plays in the pathogenesis of the disease, and specifically in the mechanisms of virus entry into the host cells of the respiratory system. Moreover, the substrate (Angiotensin I) and the product (Angiotensin 1,7) of the ACE2 enzyme is reported to be at an imbalance in other forms of acute respiratory distress syndrome [29], while low levels of Angiotensin 1,7 have been reported to be associated with the development of severe COVID-19 [30]. However, technical factors, such as rapid degradation of angiotensin peptides, make measuring these clinically challenging, thus we sought to measure ACE2, along with TMPRSS2.

The major finding stemming from our study is that serum ACE2 levels that are higher than a cutoff of 7.8 ng/mL early in the disease course are significantly associated with the need for MV. The same is true for the association of higher ACE2 levels with additional adverse outcomes, including the need for inotropic support, AKI and RRT.

Several recent studies have sought to evaluate the predictive value of serum ACE2 levels in patients with COVID-19, however, these did not specifically focus on the risk of respiratory deterioration requiring intubation and MV, which is a crucial point of clinical decision making. Furthermore, to our knowledge, no study has as yet investigated serum TMPRSS2 levels as a potential biomarker in COVID-19. Kragstrup and colleagues recently assessed ACE2, but not TMPRSS2, in hospitalized patients with COVID-19, and reported that higher baseline plasma ACE2 levels were significantly associated with poorer overall outcome (defined by worse WHOmax category) at 28 days [31]. Notably, investigators found a similar, albeit less pronounced, association among hospitalized COVID-19 negative patients who served as the control cohort. Reindel-Schwaighofer et al. compared serum ACE2 levels at two timepoints (0–3 days, and 9–11 days) between severe and non-severe COVID-19 patients and found that only the later sample correlated with disease severity [32]. They also noted a sevenfold increase in ACE2 levels in the severely affected group. An additional longitudinal study showed higher ACE2 levels in COVID-19 patients with more severe disease and further demonstrated that elevated levels persisted to a median of 114 days post-infection [33]. Most recently, Fagyas and colleagues evaluated serum ACE2 activity in severely- or critically-ill patients with COVID-19 and in non-COVID-19 severe sepsis patients and concluded that baseline ACE2 activity independently indicated COVID-19 severity and that higher ACE2 activity predicted a higher risk for 30-day mortality [34].

Unlike these studies, Rieder and colleagues compared COVID-19 positive and negative adult patients and failed to find significant differences in mean serum ACE2 concentrations [35]. Another study reported serum ACE2 levels were comparable to those seen in a healthy population, although the study sample was small (n = 13) [36].

Heterogeneity between these study results could be attributed to the individual characteristics of included patients. Indeed, differences in baseline ACE2 levels could play a significant role. Moreover, variability in staging/severity of illness at the time of measure such that comparisons, such as those by Rieder et al. only comparing COVID-19 positive patients versus negative controls, may not detect changes in the concentration related to disease severity. Finally, differences in assay sensitivity could also be contributing factors.

There is still some uncertainty regarding the net effect of ACE2 in the context of COVID-19, which makes interpreting the heterogeneous results on this subject challenging. On the one hand, its role in viral entry into host cells is well established. So much so that in the early stages of the COVID-19 pandemic, anti-ACE2 compounds were being considered as a potential therapeutic intervention. On the other hand, ACE2 is a counter-regulatory enzyme of the RAAS system and is responsible for limiting angiotensin-2 mediated pulmonary capillary leak and inflammation, as well as activating anti-inflammatory and anti-thrombotic pathways. Thus, it has been suggested to harbor a lung protective effect [37]. Furthermore, human recombinant soluble ACE2 has been used to treat COVID-19, with the rationale of binding the viral spike protein and increasing angiotensin 1,7 levels and is currently undergoing clinical trials [38]. One hypothesis would be that the higher ACE2 levels reflect higher tissue expression of ACE2, enhancing SARS-CoV-2 binding, increasing viremia, and ultimately, leading to an increased risk of poor disease progression. Indeed, many comorbidities associated with increased risk of mortality in COVID-19, such as obesity, diabetes, and heart failure, are associated with increased levels of ACE2 [39,40]. However, one could argue that the increased ACE2 could be beneficial by blunting angiotensin II-mediated inflammatory damage. It is also possible that serum ACE2 levels do not reflect tissue expression. Increased circulatory ACE2 concentration could reflect increased membrane cleavage, with subsequently decreased tissue expression, leading to decreased local tissue levels of anti-inflammatory Angiotensin 1,7 and higher levels of the pro-inflammatory angiotensin II, thus promoting end organ injury. This is supported by the SARS-CoV-2 spike protein effect of decreasing ACE2 expression in endothelial cells, which results in mitochondrial dysfunction, impaired endothelial function, and increased inflammation [41]. As such, increased ACE2 could represent enhanced membrane cleavage and maladaptive response to the virus. Taken together, either hypothesis could explain the significant association of elevated serum ACE2 concentration with the need for MV, as well as the development of AKI, as observed in this study. Given what is known in regard to the physiology of RAAS, it is unlikely that circulating serum ACE2 itself is harmful or contributes to the pathology of COVID-19 but is most likely reflective of underlying tissue related injury. The observations of this study do not thus negate the potential beneficial use of soluble ACE2 as a potential therapeutic agent in SARS-CoV-2 infection.

Of note, we found significant correlations between serum ACE2 concentrations, and values of a multitude of laboratory tests, including biomarkers of inflammation, liver function and coagulation (Supplementary Table S4). This suggests that ACE2 levels correlate with pathological pathways underlying disease severity [2,42].

As for TMPRSS2, univariate analysis showed a statistically significant association of high serum levels early in the disease course (using a cutoff of 2 ng/mL) with the risk of respiratory deterioration requiring intubation and MV (*p* = 0.005). However, this association was lost in multivariate analysis. As this work was designed as a pilot study, it is possible that larger cohorts might yield statistically significant results regarding serum TMPRSS2 levels as a potential early biomarker for risk stratification in COVID-19.

This study has some limitations. First, the sample size was relatively small, with 65 patients for whom an early (day 0) serum sample was available for ACE2 level analysis. Intended as a pilot study, the results should be interpreted as such, and further studies evaluating the association of ACE2 and TMPRSS2 levels and disease course and outcome in larger cohorts of COVID-19 patients would be needed as external validation of our findings. Second, we did not compare our cohort to a control group of COVID-19 negative patients, nor were we able to analyze serum samples obtained later in the course of the disease, beyond the second week of hospitalization. Nonetheless, the study design was sufficient for the intended purpose of evaluating whether serum levels of these two enzymes shortly after hospital admission can serve as early biomarkers and correlate with the need for MV.

## 5. Conclusions

We show that elevated serum ACE2 levels early in the course of hospitalization are associated with adverse outcomes in COVID-19 patients, including respiratory failure necessitating the need for MV. In contrast, we found no association between TMPRSS2 and disease progression. Thus, measuring serum ACE2 at hospital admission may be useful for predicting the risk of progressing toward severe disease. Larger studies are needed to corroborate these preliminary findings.

## Figures and Tables

**Table 1:** Demographic and clinical features of COVID-19 patients who required intubation and mechanical ventilation compared to those who did not.

	Non-MV N = 42	MV N = 30	*p* Value
Age *	68.93 ± 13.90	58.67 ± 13.33	**0.008**
Male	30 (71.4%)	23 (76.7%)	0.619
Weight * (N = 61)	81.73 ± 16.92	86.03 ± 18.43	0.622
Diabetes	14 (33.3%)	14 (46.7%)	0.253
HTN	25 (59.5%)	14 (46.7%)	0.280
Dyslipidemia	13 (31.0%)	14 (46.7%)	0.175
Lung disease **	3 (7.1%)	2 (6.7%)	0.938
IHD	9 (21.4%)	3 (10.0%)	0.200
CRF	8 (19.0%)	0 (0.0%)	**0.011**
CVA	2 (4.8%)	1 (3.3%)	0.765
Anemia	6 (14.3%)	3 (10.0%)	0.588
Malignancy	2 (4.8%)	1 (3.3%)	0.557

* Mean ± SD, ** Lung disease (COPD, chronic lung disease, asthma). CRF, chronic renal failure; CVA, cerebral vascular event; HTN, hypertension; IHD, ischemic heart disease; MV, mechanical ventilation. *p* values that reach statistical significance appear in bold.

**Table 2:** Clinical outcomes of COVID-19 patients with high (>7.8 ng/mL) and low ACE2 levels (<7.8 ng/mL) (as per ROC analysis).

	ACE2-First Sample <7.8 ng/mL N = 42	ACE2-First Sample >7.8 ng/mL N = 23	*p* Value
Mortality	7 (16.7%)	4 (17.4%)	0.941
Mechanical ventilation	11 (26.2%)	12 (52.2%)	**0.036**
Hospitalization > 7 days	28 (66.7%)	15 (65.2%)	0.906
Inotropic support	10 (23.8%)	12 (52.2%)	**0.021**
Acute renal failure *	11 (26.2%)	9 (39.1%)	0.280
RRT	2 (4.8%)	6 (26.1%)	**0.012**
ECMO	4 (9.5%)	3 (13%)	0.662
Blood products	12 (28.6%)	9 (39.1%)	0.384
DIC	3 (7.1%)	0 (0%)	0.189
PE/DVT	2 (4.8%)	4 (17.4%)	0.093
Bleeding	6 (14.3%)	9 (39.1%)	**0.023**
Pneumothorax	1 (2.4%)	1 (4.3%)	0.661
Sepsis	12 (28.6%)	10 (43.5%)	0.225

* Acute renal failure—increase in creatinine from baseline in 50%, DIC, Disseminated intravascular coagulation; DVT, Deep vein thrombosis; ECMO, extracorporeal membrane oxygenation; HIT, Heparin-induced thrombocytopenia; PE, Pulmonary embolism; RRT, renal replacement therapy. *p* values that reach statistical significance appear in bold.

**Table 3:** ACE2 in early disease stages is an independent predictor of the need for mechanical ventilation.

Variables	OR	95% CI	*p* Value
ACE2 (above 7.8 vs. below 7.8)	7.49	1.51–37.11	**0.014**
Age	0.89	0.83–0.95	**0.001**
Sex (Female vs. Male)	0.71	0.14–3.56	0.677
Acute renal failure *	17.68	3.13–99.86	**0.001**

* Acute renal failure—increase in creatinine from baseline in 50%, ACE2, Angiotensin converting enzyme 2; CI, confidence interval; OR, odds ratio. *p* values that reach statistical significance appear in bold.

## Data Availability

The data that support the findings of this study are available on request from the corresponding author. The data are not publicly available due to privacy or ethical restrictions.

## Supplementary Materials

The following supporting information can be downloaded at: https://www.mdpi.com/article/10.3390/jpm12040622/s1, Figure S1: ACE2 and TMPRSS2 serum levels at presentation for patients who ultimately required mechanical ventilation (MV) and those who did not (no MV); Figure S2: ROC curves for ACE2 and TMPRSS2 levels early in COVID-19 for correlation with need for mechanical ventilation; Table S1: Association between early sample ACE2 or TMPRSS2 serum expression and clinical characteristics in COVID-19 patients; Table S2: Clinical outcomes in COVID-19 patients who required mechanical ventilation compared to those who did not; Table S3: Laboratory results in COVID-19 patients who required mechanical ventilation and those who did not; Table S4: Correlations between ACE2 and other biomarkers associated with disease severity in COVID-19; Table S5: TMPRSS2 at an early disease stage is not an independent predictor of mechanical ventilation in COVID-19; Table S6: ACE2 and TMPRSS2 serum levels late in the disease course in COVID-19 patients; Table S7: ACE2/TMPRSS2 ratio does not predict the risk for mechanical ventilation in COVID-19 patients.
