# Supplementary material for: The Predictive Value of Serum ACE2 and TMPRSS2 Concentrations in Patients with COVID-19—A Prospective Pilot Study
Source: J Pers Med. 2022 Apr 12;12(4):622. doi: 10.3390/jpm12040622 (PMC9032089; doi:10.3390/jpm12040622)
Supplement: Supplementary file 1 [file jpm-12-00622-s001.zip › jpm-1637077-supplementary.pdf]

Supplemental Information Table Of Content:

SUPPLEMENTAL INFORMATION TABLE OF CONTENT: 1

SUPPLEMENTAL FIGURES AND TABLES 2

FIGURE S1 3
FIGURE S2 4
TABLE S1 4
TABLE S2 5
TABLE S3 7
TABLE S4 8
TABLE S5 9
TABLE S6 10
TABLE S7 11

Supplemental figures and tables

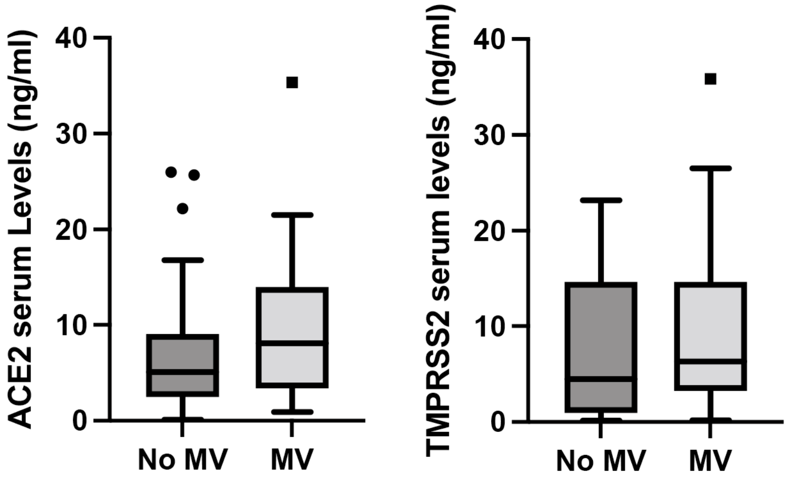

Figure S1. ACE2 and TMPRSS2 serum levels at presentation for patients who ultimately required mechanical ventilation (MV) and those who did not (no MV).

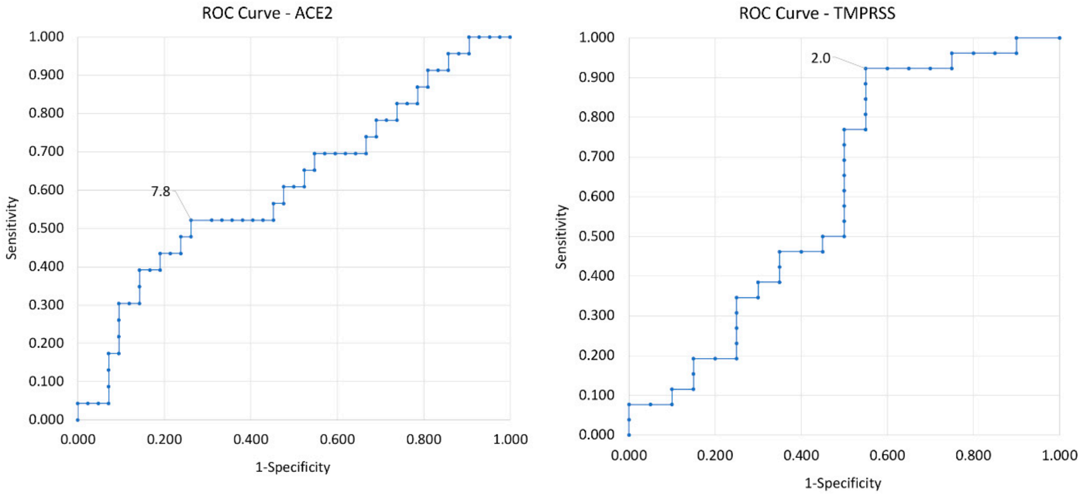

Figure S2. ***ROC curves for ACE2 and TMPRSS2 levels early in COVID-19 for correlation with need for mechanical ventilation.*** ROC analysis dichotomized ACE2 levels in order to predict the risk for requiring mechanical ventilation. ACE2 levels that produced the highest sensitivity & specificity for predicting the need for mechanical ventilation was above 7.8ng/mL. For TMPRSS2, expression levels that produced the highest sensitivity & specificity for predicting the need for mechanical ventilation was above 2ng/mL.

Table S1. ***Association between early sample ACE2 or TMPRSS2 serum expression and clinical characteristics in COVID-19 patients***

Variable		ACE2	p-value	TMPRSS2	p-value
**Hypertension**	Yes	6.24 (2.92-12.39)	0.602	6.93 (2.13-15.13)	0.698
	No	5.59 (3.60-9.99)		5.52 (2.59-13.55)	
**Diabetes**	Yes	6.60 (3.50-12.56)	0.446	6.07 (2.07-15.62)	0.572
	No	5.27 (2.44-10.01)		5.81 (2.24-13.95)	
**Dyslipidemia**	Yes	9.15 (5.66-15.80)	**0.002**	8.34 (3.73-16.28)	0.099
	No	4.13 (2.45-7.95)		4.75 (1.80-13.62)	
**Sex**	Female	5.27 (3.06-12.11)	0.976	3.58 (1.04-14.10)	0.549
	Male	5.67 (3.01-10.13)		6.33 (2.43-14.46)	

Table S2. ***Clinical outcomes in COVID-19 patients who required mechanical ventilation compared to those who did not***

	Non-ventilation n=42 n (%)	Ventilation n=30 n (%)	p value
**Mortality**	4 (9.5%)	11 (36.7%)	0.005
**Hospitalization >7 days**	20 (47.6%)	30 (100.0%)	<0.001
**Inotropic support**	0 (0.0%)	29 (96.7%)	< 0.001
**Acute renal failure***	7 (16.7%)	17 (58.6%)	< 0.001
**RRT**	0 (0.0%)	10 (33.3%)	< 0.001
**ECMO**	0 (0.0%)	8 (26.7%)	< 0.001
**Blood products**	5 (11.9%)	20 (66.7%)	< 0.001
**DIC**	0 (0.0%)	3 (10.0%)	0.036
**PE/DVT**	1 (2.4%)	7 (23.3%)	0.005
**HIT**	0 (0.0%)	3 (10.0%)	0.036
**Bleeding**	1 (2.4%)	18 (60.0%)	< 0.001
**Pneumothorax**	0 (0.0%)	3 (10.0%)	0.036
**Sepsis**	2 (4.8%)	26 (86.7%)	< 0.001

*Acute renal failure – increase in creatinine from baseline in 50%.

DIC, Disseminated intravascular coagulation; DVT, Deep vein thrombosis; ECMO, extracorporeal membrane oxygenation; HIT, Heparin-induced thrombocytopenia; PE, Pulmonary embolism; RRT, renal replacement therapy.

Table S3. ***Laboratory results in COVID-19 patients who required mechanical ventilation and those who did not***

	No mechanical ventilation N=42	Mechanical ventilation N=30	p value
**Creatinine mg\dl (Max) (N=72)**	0.98 (0.80-1.77)	1.81 (1.07-3.44)	0.003
**WBC K\micl (N=71)**	5.62 (4.59-7.27)	8.75 (6.53-13.84)	0.002
**WBC K\micl (Min) (N=71)**	5.19±1.82	5.45±2.12	0.516
**WBC K\micl (Max) (N=71)**	8.19 (5.95-11.30)	19.98 (13.96-33.20)	<0.001
**% Lympho (N=71)**	16.45 (14.15-21.30)	9.90 (6.30-15.80)	0.005
**Hb g\dl (Min) (N=72)**	12.22 (9.83-13.09)	7.08 (6.78-8.26)	<0.001
**PLT K\micl (Min) (N=72)**	161.5 (144.5-192)	118.5 (67.2-164.7)	0.005
**CRP g\dl (N=71)**	70.8 (24.6-126.6)	114.9 (70.5-243.5)	0.013
**CRP g\dl (Max) (N=72)**	107.8 (67.4-210.2)	329.7 (285.0-401.0)	<0.001
**PCT µg\l (N=41)**	0.83 (0.47-1.79)	0.47 (0.32-0.92)	0.147
**PCT µg\l (Max) (N=47)**	1.09 (0.54-3.28)	4.56 (1.88-14.27)	0.004
**ALT U\l (N=71)**	24.5 (17-32.7)	41.0 (27.0-58.0)	<0.001
**AST U\l (N=71)**	35.5 (28.2-50.5)	60.0 (42.0-95.0)	<0.001
**LDH U\l (N=71)**	288.5 (227.2-398.0)	449.0 (357.0-676.0)	<0.001
**Triglycerides mg\dl (N=38)**	148.0 (113.5-185.8)	187.0 (143.8-289.0)	0.035
**Troponin ng\ml (N=66)**	9.5 (4.7-13.7)	13.2 (9.8-36.5)	0.015
**D-dimer ng\ml (N=62)**	695.0 (495.0-1200.0)	1425.5 (649.5-3682.5)	0.021
**D-dimer max ng\ml (N=69)**	1007.0 (573.2-5569.8)	22726 (8195-47938)	<0.001
**Fibrinogen mg\dl (Min) (N=61)**	439.35±170.39	231.83±131	<0.001
**CPK U\l (N=42)**	106.5 (61.0-227.0)	162.5 (64.0-272.2)	0.930
**TNF pg\ml (N=15)**	22 (17-38.2)	28 (24.5-38)	0.571
**TNF pg\ml (Max) (N=31)**	29.5±22.27	31.44±12.43	0.844
**IL8 pg\ml (N=15)**	105 (81-262.2)	52 (33-94.5)	0.104
**IL8 pg\ml (Max) (N=31)**	75 (36.7-114)	56 (43-97)	0.783
**IL 1B pg\ml (N=15)**	0.5 (0.0-1.0)	1.0 (0.0-1.0)	0.721
**IL 1B pg\ml (Max) (N=31)**	0.00 (0.00-0.75)	1.00 (0.00-1.00)	0.383
**IL6 pg\ml (N=15)**	48.0 (34.7-65.5)	186.0 (79.0-369.0)	0.078
**IL6 pg\ml (Max) (N=32)**	36.0 (26.5-60.5)	193.0 (86.0-657.0)	0.001

Mean±SD; values upon admission if not stated otherwise. Abbreviations: WBC – White blood count, Lympho - Lymphocytes, Hb – Hemoglobin, PLT – Platelets, CRP - C-reactive protein, PCT – Procalcitonin, ALP - Alkaline Phosphatase, GGT - Gamma Glutamyl Transpeptidase, ALT - Alanine transaminase, AST - Aspartate Aminotransferase, LDH - Lactate Dehydrogenase, CPK - creatine phosophokinase, TNF - Tumor necrosis factor, IL8 - Interleukin 8, IL-1B - interleukin 1, beta, IL 6 - Interleukin 6

Table S4. Correlations between ACE2 and other biomarkers associated with disease severity in COVID-19

Variables Correlated with ACE2	Correlation Coefficient	p value
Creatinine mg\dl (Max) (N=65)	-0.018	0.886
WBC K\micl (N=64)	0.379	0.002
WBC K\micl (Min) (N=64)	0.378	0.002
WBC K\micl (Max) (N=64)	0.271	0.031
% Lympho (N=64)	-0.414	<0.001
Hb g\dl (Min) (N=65)	-0.118	0.349
PLT K\micl (Min) (N=65)	0.259	0.037
CRP g\dl (N=64)	0.494	<0.001
CRP g\dl (Max) (N=65)	0.302	0.014
PCT µg\l (N=35)	0.465	0.005
PCT µg\l (Max) (N=40)	0.348	0.028
ALT U\l (N=64)	0.296	0.018
AST U\l (N=64)	0.310	0.013
LDH U\l (N=64)	0.457	<0.001
Triglycerides mg\dl (N=34)	0.245	0.162
Troponin ng\ml (N=60)	0.229	0.078
D-dimer ng\ml (N=56)	0.470	<0.001
D-dimer max ng\ml (N=62)	0.293	0.021
Fibrinogen mg\dl (Min) (N=54)	-0.015	0.912
CPK U\l (N=40)	-0.093	0.566
TNF pg\ml (N=14)	0.312	0.277
TNF pg\ml (Max) (N=26)	0.196	0.337
IL8 pg\ml (N=14)	-0.081	0.785
IL8 pg\ml (Max) (N=26)	0.093	0.650
IL 1B pg\ml (N=14)	0.178	0.542
IL 1B pg\ml (Max) (N=26)	0.204	0.317
IL6 pg\ml (N=14)	0.147	0.616
IL6 pg\ml (Max) (N=27)	0.273	0.168

Table S5. ***TMPRSS2 at an early disease stage is not an independent predictor of mechanical ventilation in COVID-19***

	OR	95% CI	P-value
**TMPRSS1 (Above 2 vs Below 2)**	4.626	0.393-54.414	0.223
**Age**	0.828	0.728-0.941	0.004
**Gender (Male vs Female)**	1.819	0.163-20.261	0.626
**Acute renal failure***	38.920	2.809-539.37	0.006

*Acute renal failure – increase in creatinine from baseline in 50%

Abbreviations: OR = odds ratio, CI = confidence interval, ACE2 - Angiotensin converting enzyme 2

Table S6. ***ACE2 and TMPRSS2 serum levels late in the disease course in COVID-19 patients***

	ACE2 – second sample		p value	TMPRSS2 – second sample		p value
	**No** mean±SD (n)	**Yes** mean±SD (n)		**No** mean±SD (n)	**Yes** mean±SD (n)	
**MV***	8.85 (4.23-11.92) (9)	11.54 (8.00-14.41) (21)	0.298	1.97 (0.54-4.03) (9)	6.53 (2.66-20.11) (21)	**0.045**
**LOS >7 days**		10.05 (7.60-13.81) (30)	NA		4.46 (1.32-9.96) (30)	NA
**CCCU stay**	8.85 (4.23-11.92) (9)	11.5 (8.00-14.41) (21)	0.298	1.97 (0.54-4.03) (12)	6.53 (2.66-20.11) (18)	**0.045**
**Sepsis**	9.0 (4.13-13.59) (12)	10.98 (8.12-13.72) (18)	0.626	3.79 (0.54-6.67) (15)	5.67 (1.69-18.57) (15)	0.249
**AKI***	11.92 (8.24-13.86) (15)	9.67 (5.64-13.21) (15)	0.507	4.80 (1.26-13.44) (21)	4.41 (1.34-6.77) (9)	0.806
**CVVH**	10.42 (8.09-14.41) (21)	9.67 (3.82-12.45) (9)	0.342	4.80 (1.97-10.5) (25)	2.66 (0.94-7.01) (5)	0.449
**Dialysis**	9.59 (7.47-14.41) (25)	11.54 (9.67-12.45) (5)	0.978	4.51 (1.97-8.34) (25)	1.37 (0.28-21.98) (5)	0.666
**ECMO**	11.54 (8.47-14.41) (25)	7.47 (3.82-8.00) (5)	0.101	4.03 (1.31-10.5) (20)	7.01 (4.41-7.61) (10)	0.589
**Mortality**	11.73 (8.66-14.59) (20)	8.24 (4.73-11.76) (10)	0.159	5.10 (1.80-20.58) (20)	2.94 (1.04-6.00) (10)	0.120

*MV – Mechanical Ventilation; AKI - Acute renal failure – increase in creatinine from baseline in 50%, Abbreviations: ACE2 – Angiotensin converting enzyme 2, TMPRSS2 - transmembrane serine protease 2, ARF – acute renal failure, ECMO – extracorporeal membrane oxygenation, LOS – length of stay, mechanically ventilated, CVVH – continuous veno-venous hemofiltration.

Table S7. ***ACE2/TMPRSS2 ratio does not predict the risk for mechanical ventilation in COVID-19 patients***

Outcomes		ACE2/TMPRSS2 Ratio	p-value
Intubated	Yes	0.84 (0.34-2.46)	0.477
	No	1.08 (0.68-5.35)	
AKI	Yes	1.19 (0.47-9,29)	0.261
	No	0.91 (0.34-2.05)	
Dialysis/CVVH	Yes	2.07 (0.76-7.87)	0.248
	No	1.03 (0.34-2.37)	
CCCU Admission	Yes	0.90 (0.34-2.75)	0.671
	No	1.04 (0.58-4.75)	
